# A simple and cost-effective method of DNA extraction from small formalin-fixed paraffin-embedded tissue for molecular oncologic testing

**DOI:** 10.1186/1472-6890-14-30

**Published:** 2014-07-07

**Authors:** Anthony N Snow, Aaron A Stence, Jonathan A Pruessner, Aaron D Bossler, Deqin Ma

**Affiliations:** 1 Department of Pathology, Brown University, Rhode Island Hospital, Providence, Rhode Island 02806, USA; 2 Department of Pathology, University of Iowa Hospitals and Clinics, 200 Hawkins Drive, BT6008GH, Iowa City, IA 52242, USA

**Keywords:** Genomic DNA extraction, Matrix capture, FFPE tissue, Molecular oncologic tests

## Abstract

**Background:**

Extraction of DNA from formalin-fixed, paraffin-embedded (FFPE) tissue is a critical step in molecular oncologic testing. As molecular oncology testing becomes more important for prognostic and therapeutic decision making and tissue specimens become smaller due to earlier detection of suspicious lesions and the use of fine needle aspiration methods for tissue collection, it becomes more challenging for the typical molecular pathology laboratory to obtain reliable test results. We developed a DNA extraction method to obtain sufficient quantity and high quality genomic DNA from limited FFPE tissue for molecular oncology testing using a combination of H&E stained slides, a matrix capture method and the Qiagen DNA column.

**Methods:**

Three DNA extraction methods were compared: our standard procedure of manually scraping tissue from unstained slides followed by DNA extraction using the QIAamp FFPE column (Qiagen, Valencia, CA), a glue capture method (Pinpoint Solution, Zymo Research Corp, Inc) on H&E stained slides followed by DNA extraction using either the QIAamp column or the column included with the Pinpoint kit (Zymo Research). The DNA extraction protocol was optimized. Statistical analysis was performed using the paired two-sample student’s t-test.

**Results:**

The combination of the matrix capture method with the QIAamp column gave an equivalent amount of DNA as our standard extraction method using the unstained slides and a 4.6-fold higher DNA yield than using the Zymo column included in the Pinpoint Slide Solution kit. Several molecular tests were performed and DNA purified using the new method gave the same results as for the previous methods.

**Conclusions:**

Using H&E stained slides allows visual confirmation of tumor cells during microdissection. The Pinpoint solution made removal of specific tissue from the slides easier and reduced the risk of contamination and tissue loss. This DNA extraction method is simple, cost-effective, and blends with our current workflow requiring no additional equipment.

## Background

Isolating genomic DNA (gDNA) from formalin-fixed, paraffin-embedded (FFPE) tissue is a critical step in molecular oncologic testing [1-6]. In most molecular diagnostic laboratories, this is done by cutting one or two hematoxylin and eosin (H&E) stained slides and 5-10 unstained sections, 6-10 microns in thickness, from FFPE tissue blocks. The H&E slide is reviewed by a pathologist; the area of interest is circled; and the slide is used to guide macro- or microdissection of the tissue from the unstained sections for tumor enrichment [7-10]. When resection specimens with large areas of tumor are available, this technique is facile. Increasingly though, molecular analysis from small biopsies or fine needle aspirates (FNA) is requested due to improved tolerance by patients and decreased invasiveness of the procedure. Since fewer tumor cells are available in these specimens, it can be very challenging to obtain enough DNA for testing, particularly in testing for the spectrum of epidermal growth factor [11] exons 18-21 mutations from small transbronchial lung biopsies. Additional challenges to obtain adequate genetic material, irrespective of the tissue fragment size, include contamination of tumor cells with surrounding necrosis, mucin pools, inflammatory cells, and other non-neoplastic cells. Laser capture microdissection (LCM) [12,13] is an alternative; but it requires special equipment and special training for the medical technologists. It is also cost and space prohibitive for many laboratories to invest in an LCM system.

Another obstacle during specimen processing in the molecular diagnostic laboratory is accurate microdissection of marked tumor cell clusters from unstained slides. Tumor cells present in the H&E reference slide may be decreased or absent in the deeper unstained sections leading to a falsely negative result. Accurate morphological visualization is essential in microdissection of small biopsies [14]. Several groups have explored the possibility of using histochemically stained slides for DNA extraction and have reported adverse consequences of the staining on DNA amplification [14-18]. Recently, Morikawa et al [19] extensively studied the effects of H&E staining on DNA testing and showed that neither the DNA yield nor molecular oncologic test results were affected by the stains.

The Pinpoint Slide DNA Isolation System (Zymo Research Corp, Irvine, CA) is a matrix capture method which allows lifting of tissue from the slides with ease and minimizes the potential for tissue loss and contamination from flaking of tissue during scraping of the slides (http://www.zymoresearch.com/dna-purification/genomic-dna/solid-ffpe-tissue-dna/pinpoint-slide-dna-isolation-system).

In this study, we compared our standard procedure of manually microdissecting tissue from unstained FFPE tissue on glass slides with a new method using the application of Pinpoint solution (Zymo Research Corp) on non-coversliped H&E stained slides. We also optimized the DNA column purification steps by comparing the column that came with the Pinpoint Slide Solution kit (Zymo Research Corp) and the QIAamp DNA FFPE Tissue kit (Qiagen, Valencia, CA).

## Results

### Comparison of tissue harvest and DNA extraction methods

To evaluate different tissue harvesting and DNA extraction methods, the specimens were divided into three groups. The first group used the standard method in the laboratory (U-SQ), in which unstained FFPE tissue was scraped with a razor blade followed by extraction with the QIAamp kit. The second and third groups employed deparaffinization and H&E staining of the slides followed by tissue harvest using the Pinpoint reagent. The specimen was then divided into two parts for extraction of gDNA, one half with the Qiagen kit (Pinpoint harvest with QIAamp column extraction, H-PQ) and the other half with the Zymo kit (Pinpoint harvest with Zymo column extraction, H-PZ) as described above. The tissue harvested for each test group was taken from different levels of the same region on the slide to minimize variation in cellular density.

**Figure 1:**
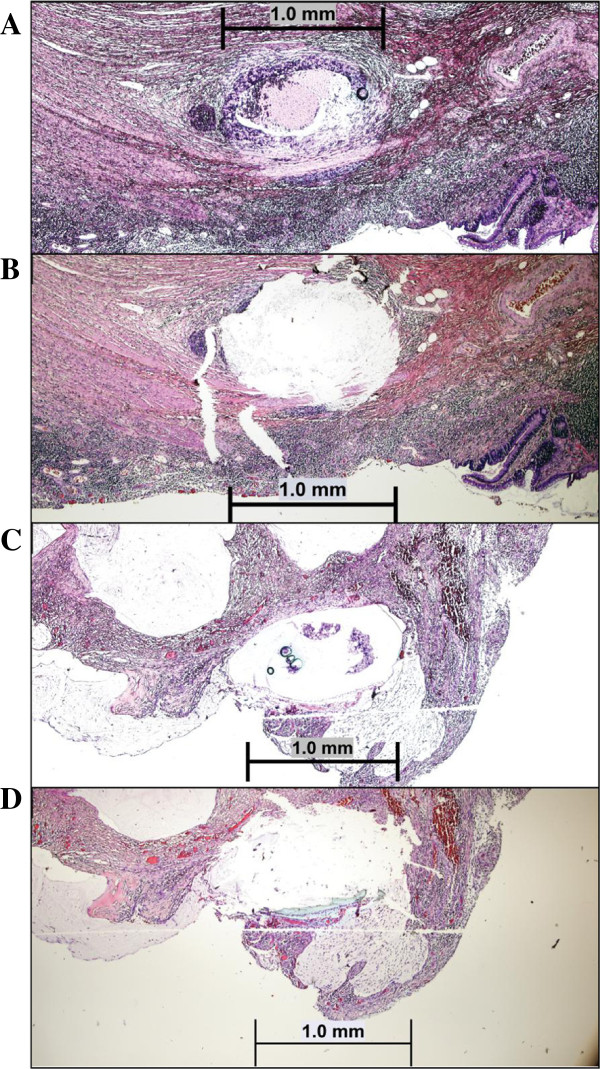
**Microdissection of H and E stained slides using matrix capture method.** Unstained slides (6 microns in thickness) were deparaffinized and H&E stained as described in Methods section. **A** and **B**: Pre- and post- removal of a 1 mm^2^ nest of adenocarcinoma cells from the pinpoint-treated section (4x); **C**: An example of clusters of adenocarcinoma cells in a mucin pool with pinpoint solution applied; **D**: The same specimen as **C** after removal of the tissue (4X).

The Pinpoint reagent was effective at obtaining the tissue from the slide. Figure 1A and B showed pre- and post-harvest of a 1 mm^2^ nest of adenocarcinoma cells from a Pinpoint-treated section. Figure 1C was an example of clusters of adenocarcinoma cells in a mucin pool with Pinpoint solution applied; and Figure 1D was the same specimen as 1C after removal of the tissue.

The quantity and quality of gDNA isolated from these methods were compared via NanoDrop spectrophotometer. The DNA yield on average from the Pinpoint matrix capture with Qiagen column purification (H-PQ; 505.0 ng/mm^2^) was not statistically different from that of the standard protocol (U-SQ, 555.0 ng/mm^2^) (Table 1). A paired student’s t-test showed a p value of .31 which provided evidence that the use of H&E staining and the Pinpoint matrix capture did not significantly decrease the yield of gDNA relative to the standard method. Subsequently, we compared the performance of the two DNA extraction columns using the H&E stained slides and Pinpoint matrix captured tissues. The yield from the Pinpoint harvest with QIAamp extraction column (H-PQ; 505.5 ng/ mm^2)^ was 4.6-fold higher than that of the Zymo column (H-PZ; 109 ng/mm^2^) and the difference was statistically significant (paired student’s t-test, p = .0005) (Table 1). DNA quality assessed by the NanoDrop spectrophotometer (A_260_/A_280_ ratio) demonstrated similar purity between the two different methods (Table 1). Longer proteinase K digestion (24-hour versus 4-hour) had no significant effect on the DNA yield (student’s t-test on the means of the DNA yield, p = 1.0). Based on the findings, H and E stain with Pinpoint matrix capture and Qiagen column purification (H-PQ) is the optimal method for tissue harvesting and DNA extraction.

**Table 1:** Comparison of different tissue harvesting and extraction methods

**Stain-Harvest/Column**	**Average DNA yield (ng/mm**^**2**^**)**	**260 nm/280 nm**
U-SQ	555.0	1.92
H-PQ	505.0	1.84
H-PZ	109.0*	1.89

The samples were divided into three test groups: U-SQ: unstained slides were scraped using a razor blade and gDNA was isolated by the Qiagen FFPE column; H-PQ: Pinpoint solution was applied to H&E stained slides, followed by extraction using the Qiagen column or Zymo column (H-PZ). See Methods for details. *Statistically significant lower yield (p = .0005).

We routinely cut 10 sections and the number of squares (1 mm^2^ in size) was recorded and the DNA yield was expressed as ng per mm^2^. Using the optimized protocol (H-PQ), we also evaluated DNA yield from a 1 mm^2^ square from a single section. When the percentage of cells in the area was close to 100%, the average DNA yield was 430 ng/mm^2^.

### Assessment of DNA amplification and mutation detection

Molecular oncology assays have varying limits of detection and sensitivity to contaminating substances. The Morikawa group [19] demonstrated that DNA obtained from H&E stained tissue did not interfere with multiple subsequent molecular testing. We assessed the ability to amplify DNA obtained from all three methods for microsatellite instability allelic discrimination and variant detection using the more sensitive primer extension assay and the less sensitive Sanger cycle sequencing. Although these methods are not quantitative, they do provide a relative assessment of mutation sequence representation within the sample.

**Figure 2:**
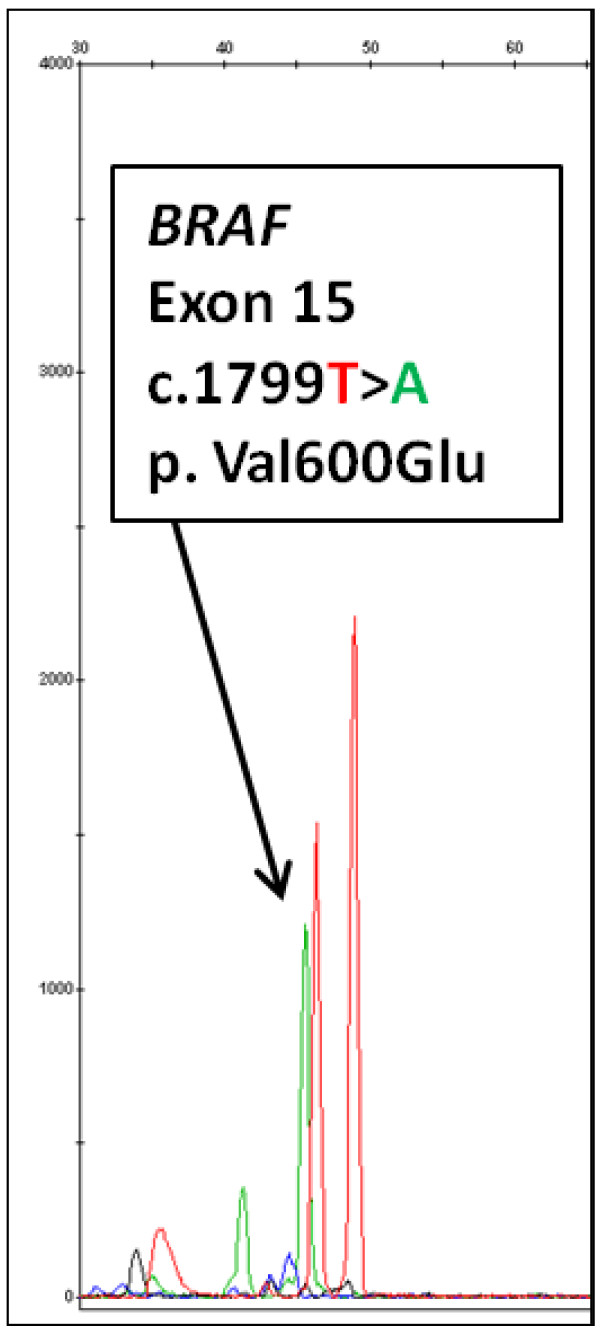
**Primer extension assay for *BRAF *V600 mutation analysis.** The primer extension assay was performed on a specimen that was shown to be positive for *BRAF* V600E mutation previously. A *c.1799 T > A*, p.V600E mutation was successfully identified using DNA extracted by the new method (Pinpoint solution was applied to H&E stained slides, followed by extraction using the Qiagen column, H-PQ). Red peak is the wild-type (nucleotide T) and green peak indicated by the arrow is the mutant peak (nucleotide A).

**Figure 3:**
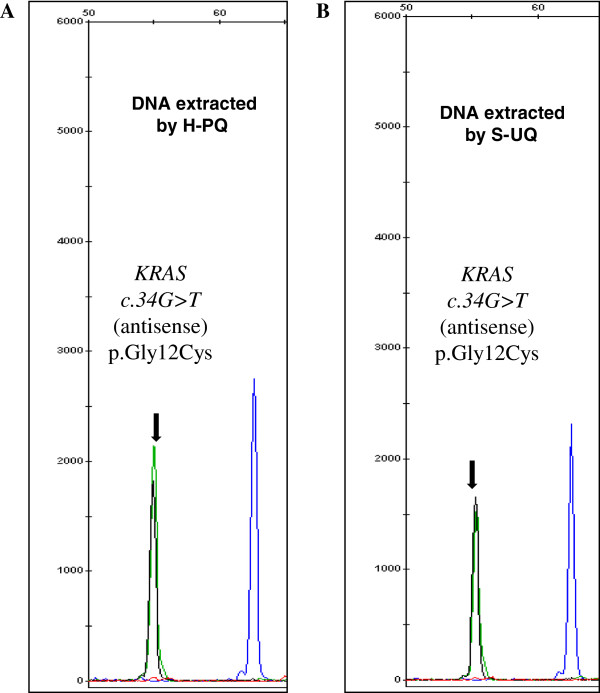
**Primer extension assay for *KRAS *codons 12, 13, and 61 mutation analysis.** DNA obtained from the new extraction method (Pinpoint solution was applied to H&E stained slides, followed by extraction using the Qiagen column, H-PQ) was used in a multiplex primer extension assay for detecting codons 12, 13, and 61 mutations (representative specimen size and microdissection shown in Figure 1A and B). **A**. *KRAS c.34G > T*, p.G12D mutation was identified in a case of colon cancer using DNA extracted by the new method (H-PQ). **B**. DNA extracted using the traditional extraction method (U-SQ) from the same specimen. Black peak is the wild type (nucleotide C) and the green mutant peak (nucleotide T) is indicated by the arrows. An antisense probe was used in this test.

DNAs obtained from the H&E stained slides followed by the Pinpoint matrix capture and QIAamp column purification were used to perform various molecular oncology assays. Multiplex primer extension assays were performed to detect the *BRAF c.1799 T > A* (p.V600E) mutation in melanomas (n = 7), colorectal carcinomas (n = 2), papillary thyroid carcinoma (n = 1) and a case of Langerhans histiocytosis. Mutations in *KRAS* codons 12, 13 and 61 were tested similarly (n = 19) (representative specimen size and microdissection shown in Figure 1A and B). Figure 2 showed a *BRAF c.1799 T > A*, p.V600E mutation in a case of malignant melanoma. The mutant peak (green peak) was about 45% of the wild-type peak, which was similar to the result using DNA extracted by the previous U-SQ method (data not shown). Figure 3 showed a *KRAS c.34G > T*, p.G12D mutation detected in a metastatic colon cancer by primer extension assay using DNA extracted by the new method (H-PQ) (Figure 3A) and the previous method using unstained slides (U-SQ) (Figure 3B).

**Figure 4:**
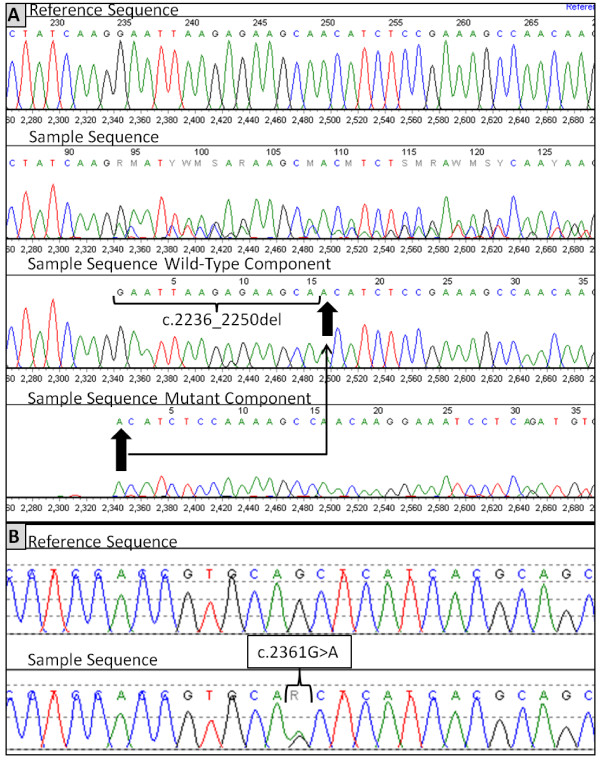
**Sanger cycle sequencing for *EGFR *mutation analysis.** Sanger cycle sequencing for *EGFR* exons 18-21 was performed on DNA obtained with the new extraction method (H-PQ, see Methods for details). **A**: Electrophoretograms showing a 15-bp deletion in exon 19 (*c.2236_ 2250del15*, p.Glu764_Ala750del) of the *EGFR* gene. Arrows marked the starting and ending positions of the deletion. **B**: A heterozygous silent mutation in exon 20 (*c.2361G > A,* p.Q787Q).

Sanger cycle sequencing, which was the least sensitive assay, was performed on 23 cases of lung adenocarcinomas. The electrophoretograms in Figure 4 showed successful detection of a 15-bp deletion in exon 19 (*c.2236_2250del15*, p.Glu764_Ala750del) (Figure 4A) and a heterozygous silent mutation in exon 20 (*c.2361G > A*, p.Q787Q) (Figure 4B).

**Figure 5:**
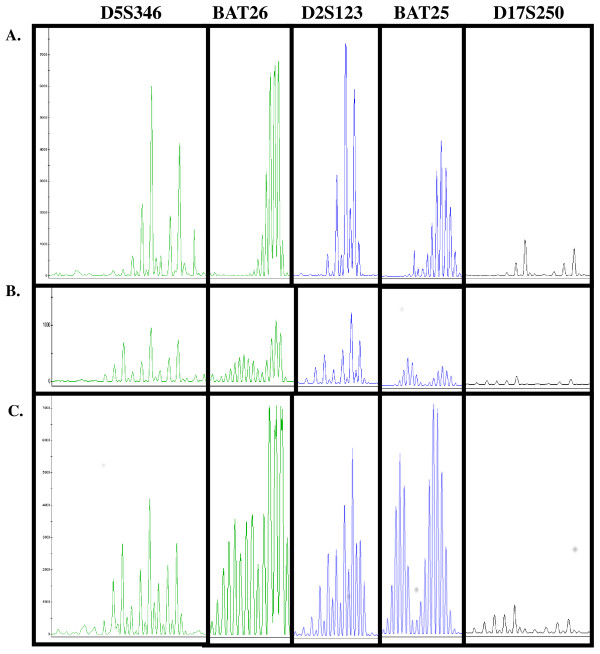
**Microsatellite instability testing (MSI) for colorectal mucinous adenocarcinoma*.*** MSI testing was performed using the 5-marker panel recommended by the National Cancer Institute. The laboratory routinely used 500 ng gDNA for this test. Similar peak height was obtained with 10-fold less DNA isolated by the newly developed extraction method (Figure 5). **A**: normal tissue; **B**: using tumor DNA extracted by the traditional method (U-SQ); **C**: using tumor DNA extracted by the new method (H-PQ).

As a proof of concept, MSI testing was performed on a mucinous adenocarcinoma of the colon using the five-marker panel (*D2S123, D5S346, D17S 250, BAT25* and *BAT26)* recommended by the National Cancer Institute. The amplification products were assessed by capillary electrophoresis (Figure 5). Comparable ratio of tumor and normal peak height was observed with DNA obtained by the new method (H-PQ) and the standard method (U-SQ).

## Discussion

FFPE tissue is the most commonly used source for molecular oncology tests [20]. Obtaining gDNA of sufficient quality and quantity from FFPE tissue is a critical step in ensuring reliable test results. This process involves selecting the right tissue block, marking area(s) with the highest tumor content and least contaminating materials by a pathologist, and microdissection of the marked area to enrich tumor content. There are multiple methods available for gDNA extraction from FFPE tissues from the traditional phenol-chloroform extraction [21-23] to column purifications using either filter or silica-based matrix [24-26]. Extraction of DNA from cellular tumors with a solid growth pattern and little contaminating, non-neoplastic tissue is relatively easy, as with gastrointestinal stromal tumors for identification of the *KIT* mutations. In contrast, increasing numbers of tests are requested on small biopsies which usually contain small nests of tumor cells surrounded by non-tumor tissue creating a challenge in obtaining adequate material. Identifying small areas with tumor on unstained slides adds a layer of uncertainty to the process.

Traditionally, most clinical laboratories use unstained slides for DNA extraction and H&E stained slides only as a reference. Morikawa’s group [19] improved the specimen processing significantly by assessing the DNA quality and assay performance of over 1000 H&E stained FFPE cases. They showed that H&E stain had no effect on DNA extraction. Based on their findings, in this study, we H&E stained all the sections in the experimental groups. Each section was examined by a pathologist and the areas with tumor were circled on the back of the slides.

H&E stain allowed us to visualize the presence of tumor cells on the slides during microdissection and reduce the risk of false negative results. In a significant portion of the cases, changes in tumor location and/or content were observed (data not shown). In fact, some laboratories routinely stain the first and last sections of the tissue block in order to minimize the false negative result. A drawback for H&E stained sections is the tissue tends to flake off in a dry powder while scraping. We therefore explored the Pinpoint matrix capture solution (Zymo Research Corp) for tissue harvest. The Pinpoint solution kept the cells together and allowed easy harvesting of minute tumor clusters surrounded by non-neoplastic cells. It also minimized potential contamination from tissue flakes in the work area and loss of valuable tumor tissue.

The advance in high throughput technology such as massively parallel sequencing [27-29] and the multiplexed genotyping method developed by Sequenom (Sequenom Inc., San Diego, CA) [30,31] allows multiple tests to be run at the same time with much less input DNA. DNAs obtained by our new extraction method worked well on the Ion Torrent AmpliSeq™ Cancer Hotspot Panel v2 (Life Technologies, Carlsbad, CA, data not shown). Both NGS and the Sequenom platforms require extensive test validation, strong bioinformatic support and a reasonable test volume to bring them on board. In small and medium sized clinical laboratories, Sanger sequencing and other low-throughput methods, which requires more input DNA, are still and will be used for oncology testing for a period of time. Our newly developed DNA extraction method allows us to obtain sufficient DNA from minute foci of tumor (1-2 mm^2^).

Test performance with DNA isolated with the new method was evaluated. Equivalent performance was observed on all assays tested in comparison with using DNA extracted by the standard method in the laboratory. Aberrations detected included *BRAF* V600E mutation in melanomas, colon cancer and histiocytosis by primer extension assay, deletion and SNP in the *EGFR* gene from lung adenocarcinomas by Sanger cycle sequencing, G12D mutation in the *KRAS* gene from a metastatic colon cancer using primer extension assay, and a multiplex PCR-based assay for microsatellite instability test from a colon biopsy with clusters of signet ring cells in a pool of mucin. The new method also yield good quality DNA based on the finding that the test performance was not affected.

An additional advantage of the new method is the reduced cost. The Leica machine used in our histology laboratory can automatically deparaffinize the slides without changing the frequency of reagent replenishment. The savings came from eliminating the need for buying xylene, ethanol, centrifuge tubes, and 1 ml pipet tips which were all needed for the manual deparaffinization procedure.

## Conclusion

We developed a simple and cost-effective method for gDNA extraction from minute FFPE tissue. The combination of H&E stain with matrix capture method and the QIAamp column yielded an equivalent quantity of DNA as our standard extraction method without compromising the qualitative test results. This method also reduced the cost, blended into our work flow nicely and required no additional equipment. It is a practical method for obtaining high quality and quantity DNA from small, challenging specimens for molecular oncologic testing in the clinical laboratories.

## Methods

This is a retrospective study based on data from clinical test development. The study was approved by the Institutional Review Board of University of Iowa.

### Case selection

Cases included in this study were selected from de-identified FFPE tissues that have been previously tested in the Molecular Pathology Laboratory at our institution since October, 2012. Special attention was paid to select cases that represented the spectrum of tissue and assays used for oncology testing. Among them, there were 19 cases of colorectal carcinomas for *KRAS* testing, 23 cases of lung adenocarcinomas for *EGFR* exons 18-21 mutational analysis, 9 cases for *BRAF* V600E testing (5 melanomas, 2 colon carcinomas, 1 papillary thyroid carcinoma and 1 Langerhans histiocytosis), and 1 case of colorectal carcinoma with matched normal tissue for microsatellite instability analysis.

### Laboratory standard protocol for isolation of genomic DNA from FFPE tissue

All cases were reviewed by a pathologist and the optimal block was selected for molecular testing. One H&E stained slide along with 10 unstained sections (6 microns in thickness) were cut. Areas of interest were circled on the H&E slide by a pathologist and corresponding areas from the unstained slides were manually microdissected using a razor blade. The paraffin flakes were placed in a 1.5 ml microcentrifuge tube, deparaffinized with 1200 μL of xylene, vortexed, and centrifuged at 16,000 g × 5 min. The tissue pellet was washed with 95% ethanol twice before proceeding with the extraction. Genomic DNA was isolated from the microdissected FFPE sections using the QIAamp DNA FFPE Tissue kit (Qiagen) which will be referred to as U-SQ (Unstained slides, Scraped harvest method, Qiagen extraction column). The deparaffinized tissue was resuspended in 180 μl of Buffer ATL, followed by 50 μl of Proteinase K (600 mAU/ML) digestion at 56°C overnight till the specimen was completely lysed. The lysate was incubated at 90°C for one hour to partially reverse the formaldehyde modification of nucleic acids. After briefly centrifugation of the tube to remove drops from the inside of the lid, 200 μl of Buffer AL was added, and the mixture was vortexed for 15 seconds. 200 μl of ethanol (96-100%) was added and mixed by vertexing for 15 seconds. The mixture was incubated for 5 min at room temperature, briefly centrifuged and the entire lysate was transferred to the QIAamp MinElute column. DNA was extracted according to the manufacturer’s instruction included in the kit.

### Matrix-capture protocol

One H&E slide with cover-slip and 10 unstained sections were obtained as described above. The unstained slides were deparaffinized and H&E stained in our histology laboratory using a standard protocol on the Leica ST5020. This automated instrument submerges the slides through three changes of xylene followed by a gradient of washes with 100% ethanol, 95% ethanol, 70% ethanol, and water. After deparaffinization, the slides were processed through the H&E staining wells. No coverslip was placed. The underside of the uncover-slipped slides was marked by a pathologist and the pinpoint matrix capture solution (Zymo Research Corp) was applied to the slide and allowed to air dry. Tissue bound to the dried solution was removed from the slide using a razor blade (Figure 1). After proteinase K digestion at 55°C for 24 hours, the sample was divided into two aliquots for genomic DNA isolation either by the QIAamp DNA FFPE Tissue kit (Qiagen) as described earlier or the Pinpoint Slide DNA Isolation kit (Zymo Research Corp). Genomic DNA extraction using the Zymo kit was performed as follows: 50 μl of Extraction Buffer and 5 μl Proteinase K were added to the tube of recovered tissue. The tube was incubated at 55°C overnight, followed by heat-inactivation at 98°C for 10 minutes. The tube was placed immediately on ice, vortexed vigorously for 15 seconds and centrifuged at 16,000 g × 5 min. The supernatant was transferred to a clean tube. The DNA concentration was measured by the NanoDrop spectrophotometer (NanoDrop, Westlake Village, CA) and the quality of gDNA was assessed by the ratio of absorbance at 260 nm and 280 nm. These two methods were designated as H-PZ for H&E stained slides, Pinpoint matrix capture and Zymo Slide DNA Isolation kit and H-PQ for H&E stained slides, Pinpoint matrix capture with the Qiagen QIAamp DNA FFPE column purification.

### Comparison of tissue harvest and DNA extraction methods

The specimens were divided into three groups. The first group used the standard method in the laboratory (U-SQ). In this method, unstained FFPE tissue was scraped with a razor blade followed by extraction with the QIAamp kit as described above. The second and third groups employed deparaffinization and H&E staining of the slides followed by tissue harvest using the Pinpoint reagent. The specimen was then divided into two parts for extraction of gDNA, one half with the Qiagen kit (Pinpoint harvest with QIAamp column extraction, H-PQ) and the other half with the Zymo kit (Pinpoint harvest with Zymo column extraction, H-PZ) as described above. The tissue harvested for each test group was taken from different levels of the same region on the slide to minimize variation in cellular density.

### Molecular tests performed

#### *BRAF* codon V600 mutation analysis by primer extension assay

*BRAF* exon 15 was PCR amplified using primers 5′-GATCCCTTTACTTACTACACCTCAG-3′ and 5′-GATCGTAACTCAGCAGCATCTC-3′. Mutations in codon V600 were examined by single nucleotide primer extension assay using the Snapshot Multiplex kit (Applied Biosystems, Foster City, CA). The V600 codon was interrogated with a sense probe consisting of deoxy-thymidine monophosphate homopolymers of varied sizes for discrimination of different mutation products [32] (See reference [32] for probe sequences). After amplification, the product was subjected to probe annealing and addition of a single, fluorescently labeled dideoxynucleotide. The primer extension products were analyzed by capillary electrophoresis.

#### *KRAS* codon 12, 13, and 61 mutation analysis by primer extension assay

This assay was performed with a similar procedure to that described for the *BRAF* V600 primer extension assay above. In this assay, exons 2 and 3 of the *KRAS* gene were amplified using specific primers in a multiplex reaction (see reference 32 for primer sequences). The primer extension probes were designed with varying lengths of deoxythymidine monophosphate homopolymers ranging from 30 to 76 bases to allow for discrimination of the products by size. The sense probes were designed to end one base 5′ of the following positions: *c.35, c.38,* and *c.182*. The antisense probes were designed to end one base 5′ of the following positions: *c.34, c.37, c.57, c.181*, and *c.183*. These probes allowed for the detection of common variants present in codons 12, 13, and 61 of the *KRAS* gene.

#### *EGFR* mutation analysis by Sanger sequencing

Exons 18-21 of the *EGFR* gene were PCR amplified using primers designed to produce one amplicon per exon. Primer sequences were: 5′-TCTGGCACTGCTTTCCAGC-3′ and 5′-TCCCAAACACTCAGTGAAACAAA-3′ for exon 18; 5′-ACCCAG ATC ACTGGGCAGC-3′ and 5′-AGCAGCTGCCAGACATGAG-3′ for exon 19; 5′-CTGGCCACCATGCGAAGCC-3′ and 5′-ATCCTGGCTCCTTATCTCC-3′ for exon 20; and 5′-CCCATGATGATCTGTCCCT-3′ and 5′-TGGTCCCTGGTGTCAGGAA-3′ for exon 21. 25 to 150 ng of gDNA was used as the starting material in each amplification reaction. The PCR thermocycling conditions were as follows: 94°C for 2 minutes, followed by 30 cycles of 94°C for 15 seconds, 58°C for 30 seconds, and 72°C for 30 seconds. The amplification products were purified using the Qiagen QIAquick PCR purification kit (Qiagen). Cycle sequencing was performed using the ABI Big Dye Terminator Mix, version 3.1 and the products were separated by capillary electrophoresis on an ABI 3130 Genetic Analyzer (Applied Biosystems).

### Microsatellite instability testing by PCR followed by capillary electrophoresis

The National Cancer Institute (NCI) recommended five-marker panel (two mononucleotide markers, *BAT25* and *BAT26* and three dinucleotide markers, *D2S123, D5S346* and *D17S250*) were used for microsatellite instability testing in two multiplexed reactions with fluorescently labeled primers. Genomic DNA extracted from tumor and paired normal tissue from the same patient was used. The amplicons were then analyzed using capillary electrophoresis.

### Comparison of 4-hour versus overnight proteinase K digestion

Three samples with uniform cellularity were selected. Ten slides were prepared by deparaffinization and H&E staining without placement of coverslips. Areas with the greatest cellular uniformity were marked by a pathologist using a 2 mm × 2 mm template. The pinpoint reagent was applied and the dried matrix was removed as described above. The matrix pellet was digested with proteinase K at 55°C for 4 hours and then divided into two aliquots: one was extracted immediately using the QIAamp FFPE kit (Qiagen); the other one was subjected to continued proteinase K digestion overnight and DNA was extracted using the same column type the next day. Optical densitometry was performed after extraction and the mean concentration values were compared statistically.

### Statistical analysis

Statistical analysis was performed using the paired two-sample student’s t-test. A significant difference is defined as p value less than .05.

## Abbreviations

FFPE: Formalin-fixed, paraffin-embedded; gDNA: Genomic DNA; MSI: Microsatellite instability; U-SQ: Unstained slides, Scraped harvest method, Qiagen extraction column; H-PZ: H&E stained slides, Pinpoint matrix capture and Zymo Slide DNA Isolation kit; H-PQ: H&E stained slides, Pinpoint matrix capture with the Qiagen QIAamp DNA FFPE column.

## Competing interests

The authors declare that they have no competing interests.

## Authors’ contributions

ANS was involved in data analysis and interpretation, and drafting the manuscript. AAS carried out the molecular oncology studies and data analysis. JP helped AAS with designing and optimizing the tests. ADB helped with trouble-shooting and editing the manuscript. DM designed the study, participated in data analysis, interpretation, drafted and finalized the manuscripts. All authors agree to be accountable for all aspects of the work. All authors read and approved the final manuscript.

## Pre-publication history

The pre-publication history for this paper can be accessed here:

http://www.biomedcentral.com/1472-6890/14/30/prepub
